# Proteomic Analysis Reveals New Cardiac-Specific Dystrophin-Associated Proteins

**DOI:** 10.1371/journal.pone.0043515

**Published:** 2012-08-24

**Authors:** Eric K. Johnson, Liwen Zhang, Marvin E. Adams, Alistair Phillips, Michael A. Freitas, Stanley C. Froehner, Kari B. Green-Church, Federica Montanaro

**Affiliations:** 1 Center for Gene Therapy, The Research Institute at Nationwide Children’s Hospital, and the Ohio State University Biochemistry Program, Columbus, Ohio, United States of America; 2 Center for Biomedical EPR Spectroscopy and Imaging, the Ohio State University, Columbus, Ohio, United States of America; 3 Department of Physiology and Biophysics, University of Washington, Seattle, Washington, United States of America; 4 Division of Cardiothoracic Surgery, The Heart Institute, Cincinnati Children’s Hospital, Cincinnati, Ohio, United States of America; 5 Department of Molecular Virology, Immunology and Medical Genetics, Comprehensive Cancer Center, The Ohio State University, Columbus, Ohio, United States of America; 6 Department of Pediatrics, The Ohio State University College of Medicine, Columbus, Ohio, United States of America; King’s College, London, United Kingdom

## Abstract

Mutations affecting the expression of dystrophin result in progressive loss of skeletal muscle function and cardiomyopathy leading to early mortality. Interestingly, clinical studies revealed no correlation in disease severity or age of onset between cardiac and skeletal muscles, suggesting that dystrophin may play overlapping yet different roles in these two striated muscles. Since dystrophin serves as a structural and signaling scaffold, functional differences likely arise from tissue-specific protein interactions. To test this, we optimized a proteomics-based approach to purify, identify and compare the interactome of dystrophin between cardiac and skeletal muscles from as little as 50 mg of starting material. We found selective tissue-specific differences in the protein associations of cardiac and skeletal muscle full length dystrophin to syntrophins and dystrobrevins that couple dystrophin to signaling pathways. Importantly, we identified novel cardiac-specific interactions of dystrophin with proteins known to regulate cardiac contraction and to be involved in cardiac disease. Our approach overcomes a major challenge in the muscular dystrophy field of rapidly and consistently identifying *bona fide* dystrophin-interacting proteins in tissues. In addition, our findings support the existence of cardiac-specific functions of dystrophin and may guide studies into early triggers of cardiac disease in Duchenne and Becker muscular dystrophies.

## Introduction

**Figure 1:**
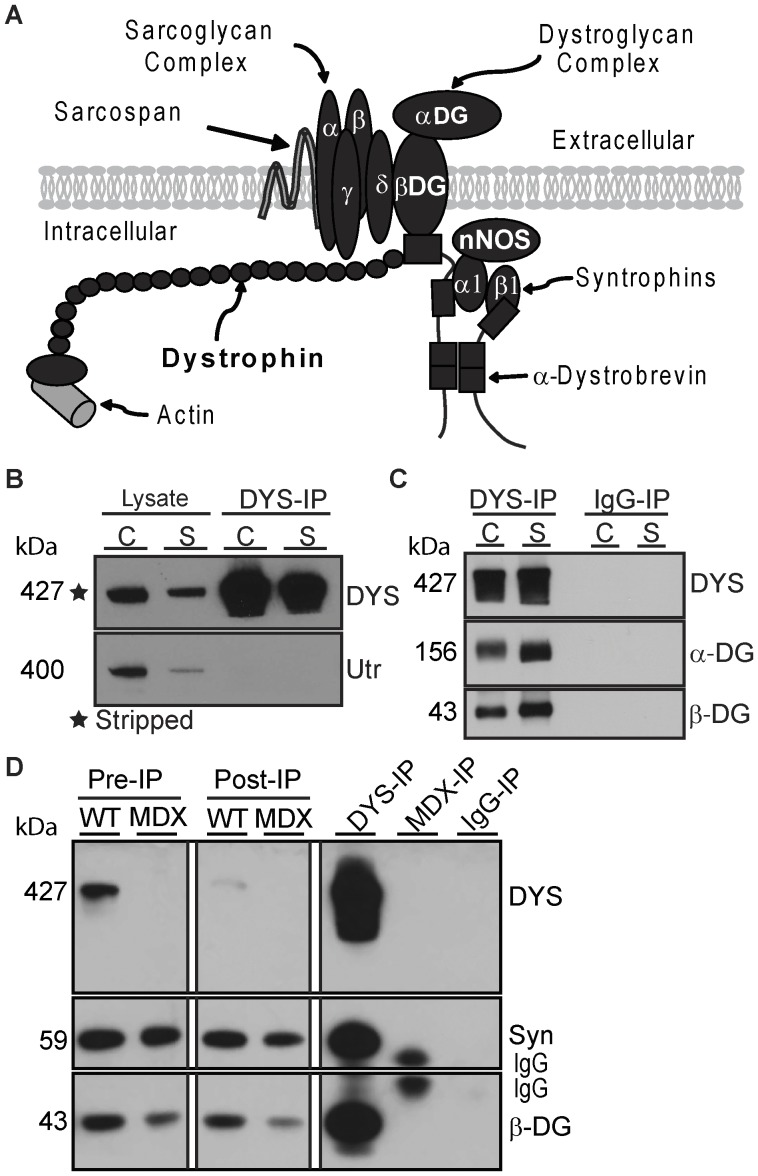
MANDYS1 specifically immunoprecipitates dystrophin and associated DAPC members. (**A**) Schematic representation of the core DAPC in skeletal muscle. (**B**) MANDYS1 does not recognize utrophin. Western blot of lysates and DYS-IPs from cardiac (C) and skeletal (S) muscle probed for utrophin (Utr), then stripped and re-probed for dystrophin. (**C**) α- and β-dystroglycan are detected in DYS-IPs but not control IgG-IPs. (**D**) Syntrophins (Syn) and β-dystroglycan (β -DG) in DYS-IP from wild type but not mdx skeletal muscle or in IgG-IPs. Dystrophin is depleted in Post-IP lysates from WT muscle.

Dystrophin is a large (427 kDa) sub-membrane protein that links the actin cytoskeleton to the extracellular matrix via the dystrophin-associated protein complex (DAPC; Figure 1A) [1]. In skeletal muscle, the DAPC has a structural role important for membrane integrity and a signaling role mediated by its intracellular members, syntrophins and dystrobrevins [2]. Mutations in dystrophin give rise to dystrophinopathies, a term that includes Duchenne muscular dystrophy (DMD), Becker muscular dystrophy (BMD) and X-linked dilated cardiomyopathy (XLDCM). DMD and BMD are characterized by both progressive skeletal muscle degeneration and cardiac involvement, contributing to early mortality by respiratory or cardiac failure [3], [4]. By contrast, XLDCM patients show a selective severe cardiac involvement leading to heart failure [5]. Although the functions of dystrophin and composition of the DAPC are generally thought to be similar between cardiac and skeletal muscle, clinical studies in dystrophinopathy patients show no correlation between cardiac and skeletal muscle disease with respect to severity or age of onset [5], [6], [7]. In addition, mini- and micro-dystrophin constructs developed for gene-replacement therapy of DMD show differences in their ability to functionally rescue cardiac versus skeletal muscle [8], [9]. These results suggest that dystrophin may have cardiac-specific functions that remain to be elucidated. Since protein interactions mediate many of the structural and signaling functions of dystrophin, we hypothesized that dystrophin may associate with different proteins in cardiac and skeletal muscle.

Mass spectrometry based proteomic approaches are well positioned for the identification of large numbers of proteins within a complex sample and could provide a comprehensive view of the dystrophin interactome. To date, proteomic analysis of muscle membrane fractions enriched for dystrophin and the DAPC has proven challenging, achieving only a 2% coverage of the large dystrophin protein and incomplete detection of known dystrophin-interacting proteins [10]. However, optimization of this approach is a worthwhile endeavor because it has the potential to reveal new tissue-specific dystrophin-binding proteins relevant to normal function and disease.

We describe here the successful combination of DAPC immunoprecipitation with shotgun proteomics (LC-MS/MS) to rapidly and consistently identify dystrophin-associated proteins from as little as 50 mg of tissue, allowing studies in individual mice and eventually biopsy material. Furthermore, LC-MS/MS yielded higher sensitivity and protein coverage than previous gel-based approaches [10], allowing robust detection of all known DAPC members with high protein sequence coverage. We further describe a spectral count analysis for subtraction of tissue-specific background and direct comparison of dystrophin’s interactome between cardiac and skeletal muscle. This analysis brought to the forefront tissue-specific differences in DAPC composition and revealed new dystrophin interacting proteins that are relevant to cardiac function and disease.

## Results

### Dystrophin Immunoprecipitation and Identification of Interacting Proteins by LC-MS/MS

To identify proteins that selectively associate with dystrophin, we opted for the high specificity of antibody-based immunoprecipitation using the MANDYS1 monoclonal antibody to dystrophin. MANDYS1 recognizes a domain not involved in interactions with known DAPC members that is absent in shorter dystrophin isoforms expressed in non-muscle cells [11], [12]. Therefore we are isolating proteins associated with full-length dystrophin expressed in muscle cells. Furthermore, MANDYS1 does not cross-react with utrophin (Figure 1B), a homolog of dystrophin [13]. Sample contamination by immunoglobulins was minimized by cross-linking the MANDYS1 antibody to the support matrix (Figure S1A, B). Western blot analysis confirmed that MANDYS1 reliably immunoprecipitated large amounts of dystrophin and co-purified transmembrane (β-dystroglycan), extracellular (β-dystroglycan) and intracellular (syntrophins) DAPC members (Figure 1C, D). LC-MS/MS analysis was performed on dystrophin immunoprecipitations from 50 mg of cardiac or skeletal muscle (quadriceps) from wild type mice (DYS-IPs). Two controls for non-specific protein binding to immunoglobulins or to MANDYS1 were performed using an irrelevant isotype-matched antibody on wild type samples (IgG-IP) or the MANDYS1 antibody on samples from dystrophin-deficient *mdx* mice [14] (MDX-IP; Figure S1C). Dystrophin and DAPC proteins were not detected in IgG- or MDX-IPs with the exception of one skeletal muscle IgG-IP that was cross-contaminated with a small amount of a cardiac DYS-IP sample resulting in low level detection of cardiac proteins and DAPC members. Comparison of IgG- and MDX-IPs revealed that they were equivalent in identifying contaminating proteins in DYS-IPs. However, different background proteins were identified in cardiac and skeletal muscle control immunoprecipitations. After tissue-specific background subtraction, dystrophin and the core DAPC members (with the exception of the more distantly associated sarcospan) were within the top 15 proteins specifically detected in all DYS-IPs with high confidence scores typically above 100, multiple unique peptide matches, and high protein sequence coverage (Table 1). This included DAPC members known to be at least one interaction removed from dystrophin, such as the sarcoglycans. Furthermore, we consistently identified and obtained good peptide coverage for transmembrane proteins (β-dystroglycan and all sarcoglycans) whose hydrophobicity renders identification by mass spectrometry challenging. Of note, two DAPC members, neuronal nitric oxide synthase (nNOS) and β2-syntrophin were consistently differentially detected between cardiac and skeletal muscle. Overall these results indicate that our approach allows for the reliable and rapid one-shot identification of proteins that interact directly or indirectly with dystrophin from as little as 50 mg of muscle tissue.

**Table 1:** Identification of DAPC members in DYS-IPs by LC-MS/MS.

			Skeletal Muscle			Heart		
Protein	UniProt ID	Expt.	Score	%	Pep.	Score	%	Pep.
				Cov.	#		Cov.	#
Dystrophin	P11531	1	19064	51.9	167	35126	63.4	207
		2	13864	42.2	125	17334	49.9	194
		3	22567	52.5	158	16027	45	149
*Dystroglycan	Q544G5	1	477	5.7	5	285	10.2	7
		2	55	3.7	2	37	2	1
		3	584	11.5	8	39	0.9	1
α-Dystrobrevin	E9QJX4	1	1388	27.7	14	2333	45.8	20
		2	926	25.5	10	572	21.1	11
		3	1674	30.9	16	522	17.6	10
nNOS	Q9Z0J4-5	1		11.3	12	–	–	–
		2	81	2.7	2	–	–	–
		3	86	1.5	2	–	–	–
α1-Syntrophin	A2AKD7	1	1610	53.5	16	2134	47.1	19
		2	1587	42.3	12	1328	62	19
		3	2429	52.9	18	1698	43.8	17
β1-Syntrophin	Q99L88	1	194	17.1	8	–	–	–
		2	–	–	–	134	10.2	4
		3	178	11.6	6	418	21	10
β2-Syntrophin	Q542S9	1	–	–	–	228	13.1	4
		2	–	–	–	356	12.3	5
		3	–	–	–	469	13.3	7
*α-Sarcoglycan	Q5SWB2	1	715	26.4	8	963	32.3	10
		2	28	3.8	1	311	17.3	5
		3	328	25.7	8	467	20.1	7
*β-Sarcoglycan	P82349	1	779	40.4	9	1074	40.4	8
		2	35	6.9	1	263	22.4	4
		3	396	23.4	5	619	24.9	5
δ-Sarcoglycan	P82347	1	548	27.1	7	1412	33.6	8
		2	559	23	6	337	19.9	6
		3	559	23	6	614	21.9	6
γ-Sarcoglycan	P82348	1	428	19.2	5	366	16.8	4
		2	201	11.3	2	310	14.4	4
		3	113	12	3	194	12	3
*Sarcospan	E9Q8Y7	1	59	6.3	1	93	6.3	1
		2	–	–	–	49	7.7	1
		3	68	6.3	1	133	14	2

Results from three independent experiments are shown for each protein. UniProt ID: UniProt protein identifier; Score: Mascot protein score; % Cov: Percent protein sequence coverage; Pep. #: Number of unique peptides with individual peptide score >30.

* For additional information for proteins identified by one unique peptide see Figures S3 and S4.

### Identification of Tissue-specific Dystrophin Protein Interactions by Multi-factor Spectral Count Analysis

To identify in an unbiased fashion both known and novel proteins that interact with dystrophin in skeletal and cardiac muscle, we adapted a multi-factor sample analysis commonly used in genomics [15] to the analysis of protein spectral counts. First, a multi-factor significance analysis test was used to account for variability between replicates and to generate a fold change and associated p-value between each DYS-IP and its corresponding IgG-IP. Negative fold changes indicated enrichment in the IgG-IP allowing easy removal of contaminating proteins from analysis. Second, proteins were sorted based on their p value to identify differences in composition and protein abundance between cardiac and skeletal muscle DYS-IPs (Table 2). In agreement with our results based on total protein scores (Table 1), the spectral count analysis identified tissue-specific differences in the associations of nNOS and β2-syntrophin with cardiac and skeletal muscle dystrophin (Table 2). In addition, comparison of p values suggested a lower abundance of β1-syntrophin in cardiac compared to skeletal muscle DYS-IPs (Table 2). Importantly, this analysis highlighted new potential dystrophin-associated proteins that were specific to cardiac DYS-IPs and involved proteins with known associations to cardiac disease in humans: Cavin-1 (PTRF) [16], [17], Ahnak1 (desmoyokin) [18], Cypher (LDB3, ZASP) [19], [20], [21], and Crystallin alpha B (CRYAB) [22], [23]. Therefore, this multi-factor analysis of spectral counts can be used to rapidly compare the composition of purified dystrophin complexes between tissues and to bring to the forefront potential candidate proteins for tissue-specific association to dystrophin.

**Table 2:** Comparison of proteins between cardiac and skeletal muscle IPs identified by multi-factor analysis of spectral counts.

		Heart spectral counts							Skm spectral counts						
		IgG-IPs			DYS-IPs				IgG-IPs			DYS-IPs			
Uniprot ID	Protein Name	1	2	3	1	2	3	p value	1	2	3	1	2	3	p value
P11531*	Dystrophin	0	0	0	1163	642	501	7.66E-08	3	0	0	621	490	917	1.09E-07
A2AKD7*	α1-Syntrophin	0	0	0	79	51	59	4.00E-05	0	0	0	55	45	105	4.38E-06
E9QJX4*	α-Dystrobrevin	0	0	0	80	25	24	1.63E-04	0	0	0	49	45	73	7.21E-06
P82347*	δ-Sarcoglycan	0	0	0	39	14	16	6.40E-04	1	0	0	18	11	36	1.45E-03
Q5SWB2*	α-Sarcoglycan	0	0	0	34	9	14	1.06E-03	0	0	0	22	2	27	6.40E-04
Q9Z0J4-5*	nNOS								0	0	0	28	5	9	8.01E-04
O54724	Cavin-1	0	0	0	14	8	35	1.01E-03							
P82349*	β-Sarcoglycan	0	0	0	27	8	17	1.17E-03	0	0	0	24	2	15	1.05E-03
P82348*	γ-Sarcoglycan	0	0	0	15	7	10	3.16E-03	0	0	0	15	6	8	1.45E-03
Q99L88*	β1-Syntrophin	0	0	0	0	5	23	1.33E-02	0	0	0	17	0	15	2.02E-03
Q542S9*	β2-Syntrophin	0	0	0	7	13	14	2.91E-03							
Q52L78	CRYAB	0	0	0	7	12	8	4.85E-03							
Q9JKS4-3	Isoform 3 of Cypher	0	0	0	3	3	13	1.13E-02							
F7BRM2	AHNAK1	0	0	0	2	2	10	1.98E-02							
Q544G5*	Dystroglycan	0	0	0	14	1	1	2.99E-02	3	0	0	13	4	28	2.09E-02

Spectral counts are shown for three independent biological replicates for DYS-IP and corresponding control IgG-IPs from cardiac and skeletal muscles. Proteins listed were enriched in DYS-IP with a p value ≤0.03.

* Known DAPC members.

### nNOS is not in a Complex with Full Length Cardiac Dystrophin

**Figure 2:**
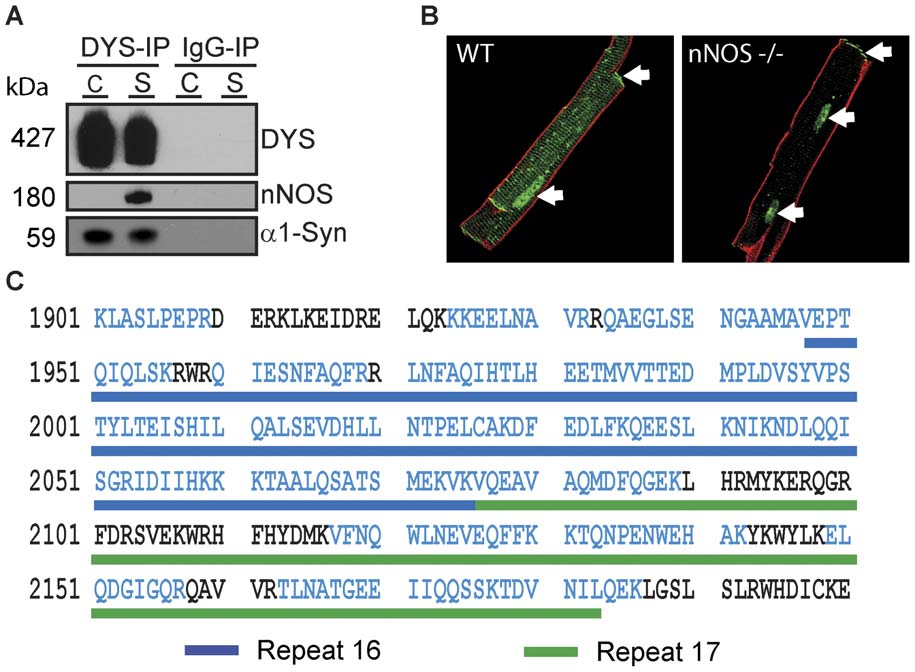
nNOS does not associate with full-length dystrophin in cardiomyocytes. (**A**) Western blot analysis of DYS-IPs and IgG-IPs from wild type cardiac (C) and skeletal (S) muscle showing lack of nNOS detection in cardiac DYS-IP but presence of α1-syntrophin. (**B**) Immunolabeling of wild type (WT) and nNOS knock-out (nNOS−/−) cardiomyocytes for nNOS (green) and dystrophin (red) shows lack of co-localization. Arrows indicate non-specific labeling. (**C**) Peptide coverage (blue amino acids) by LC-MS/MS of spectrin repeats 16 and 17 of cardiac dystrophin.

To test for tissue-specific differences in nNOS association with the DAPC, we first tested for the presence of nNOS in DYS-IPs by Western blot. As shown in Figure 2A, nNOS is readily detected in skeletal muscle dystrophin IPs, but not in cardiac DYS-IPs even when the entire DYS-IP eluate is loaded in a single lane and the film exposure is saturated. Furthermore, immunostaining of isolated cardiomyocytes showed a lack of co-localization of nNOS with dystrophin at lateral membranes (Figure 2B). Instead, nNOS localized to internal membranes consistent with its reported association with the sarcoplasmic reticulum [24]. These results support our proteomic analysis and indicate that nNOS does not interact with full length dystrophin in cardiomyocytes. Recruitment of nNOS to the DAPC occurs via alpha1-syntrophin [25] and spectrin repeats 16 and 17 in the dystrophin rod domain [26]. α1-syntrophin was readily detected in cardiac DYS-IPs by both LC-MS/MS (Table 1) and Western blot (Figure 2A). Analysis of dystrophin peptides detected by LC-MS/MS indicated that spectrin repeats 16 and 17 were present in cardiac dystrophin (Figure 2C). These results indicate that lack of association of nNOS with cardiac dystrophin is not due to loss of α1-syntrophin or to tissue-specific alternative splicing of the rod domain of dystrophin.

### Cardiac and Skeletal Muscle DAPCs Differ in Syntrophin Composition

**Figure 3:**
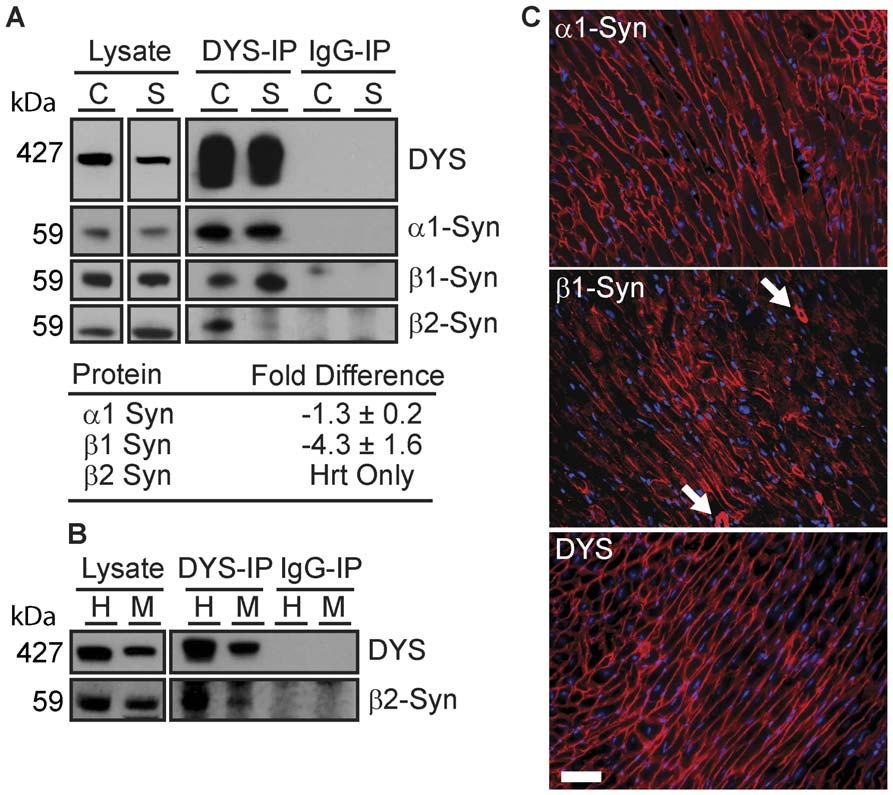
Syntrophins differ between cardiac and skeletal muscle DAPC. (**A**) Western blot analysis of syntrophins in mouse cardiac (C) and skeletal (S) muscle protein lysates, DYS-IPs and IgG-IPs. β2-syntrophin associates with dystrophin only in the heart. Fold differences in syntrophin abundance in cardiac vs. skeletal muscle DYS-IPs relative to dystrophin are shown (averages ±SD, N = 3). (**B**) Western blot analysis of DYS-IPs from human (H) and mouse (M) cardiac samples showing association of β2-syntrophin with dystrophin in the human heart. (**C**) Immunolabeling of cardiac sections from wild type mice for indicated proteins. Scale bar: 50 µm. Arrows indicate blood vessels.

LC-MS/MS analysis suggested a selective association of β2-syntrophin with cardiac dystrophin (Tables 1 and 2). Western Blot analysis confirmed co-purification of β2-syntrophin in mouse cardiac but not skeletal muscle DYS-IPs (Figure 3A). In addition, β2-syntrophin also co-purified with cardiac dystrophin in human cardiac biopsy samples (Figure 3B). Co-localization of dystrophin and β2-syntrophin by immunolabeling could not be established due to weak binding of the only available antibody to β2-syntrophin. Our spectral count analysis also indicated that α1-syntrophin is present in similar amounts in cardiac and skeletal muscle DYS-IPs while β1-syntrophin is less abundant in cardiac DYS-IPs (Table 2). Western blot analysis followed by densitometry confirmed a similar abundance of α1-syntrophin relative to dystrophin between skeletal and cardiac muscle DYS-IPs (Figure 3A). This agreed with immunohistochemical analysis of cardiac and skeletal muscle tissue sections where α1-syntrophin was strongly expressed at the membrane of all cardiomyocytes (Figure 3C) and all myofibers (Figure S2) where it co-localized with dystrophin. By contrast, 4-fold less β1-syntrophin was associated with dystrophin in cardiac muscle compared to skeletal muscle DYS-IPs (Figure 3A). This was supported by immunohistochemistry where low levels of β1-syntrophin expression were seen at the membrane of a subset of cardiomyocytes (Figure 3C), while in skeletal muscle, β1-syntrophin was expressed at high levels in a subset of muscle fibers (type IIB), as previously reported [27] (Figure S2). Overall these results confirm that cardiac and skeletal muscle DAPCs differ in the presence of β2-syntrophin and in the relative abundance of β1-syntrophin. They also validate the use of p values derived from the spectral count analysis as good indicators of the relative abundance of a given protein between samples.

### Differential Association of α-dystrobrevins with Cardiac and Skeletal Muscle DAPC

**Figure 4:**
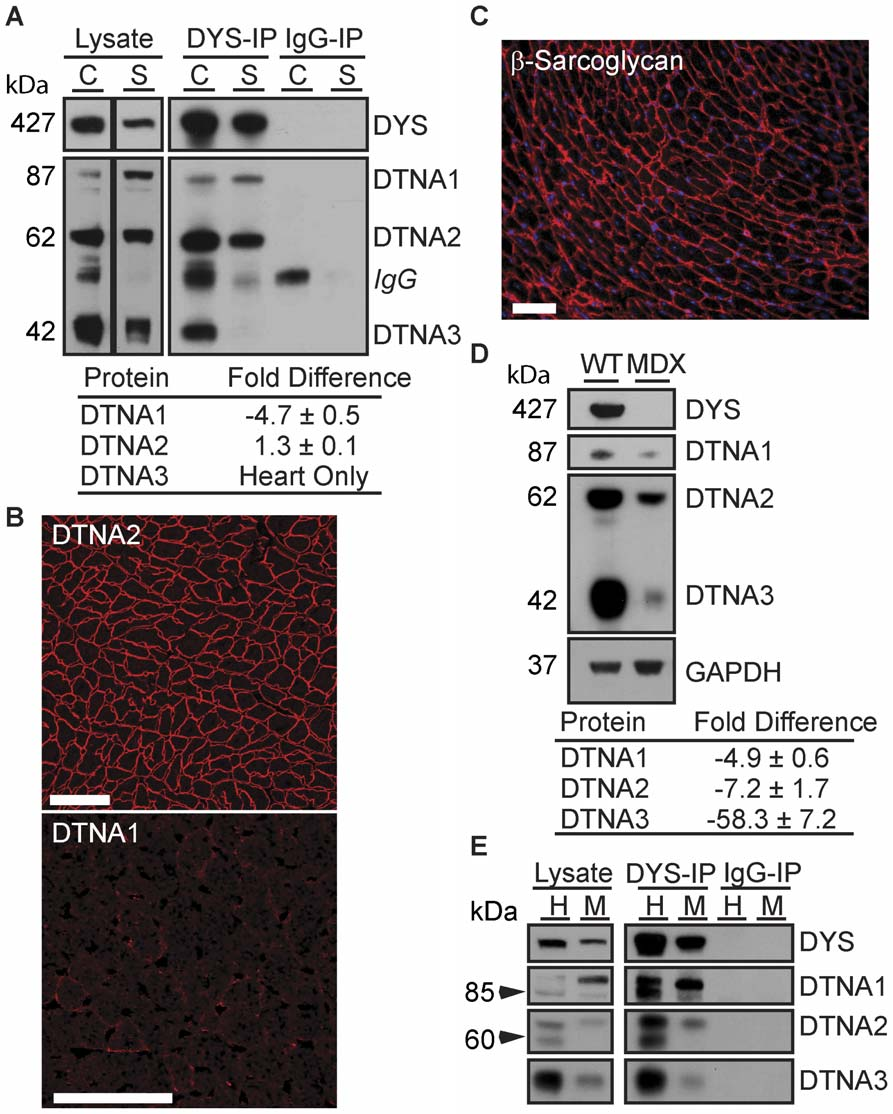
Differences in α-dystrobrevin splice variants between cardiac and skeletal muscle DAPC. (**A**) Western blot analysis of α-dystrobrevins in mouse cardiac (C) and skeletal (S) muscle total protein lysates, DYS-IPs and IgG-IPs. α3-dystrobrevin associates with dystrophin in the heart. Fold differences in α-dystrobrevin abundance in cardiac vs. skeletal muscle DYS-IPs relative to dystrophin are shown (averages ±SD, N = 3). (**B**) Immunolabeling of wild type cardiac sections for α1 and α2-dystrobrevins. Scale bar: 50 µm. (**C**) Immunolabeling of *mdx* cardiac tissue section for β-sarcoglycan. Scale bar: 50 µm. (**D**) Western blot analysis of α-dystrobrevins in heart protein lysates from wild type (WT) and *mdx* mice. Fold differences in α-dystrobrevin abundance in WT vs. mdx cardiac lysates relative to GAPDH are shown (averages ±SD, N = 3) (**E**) Western blot analysis of α-dystrobrevins in DYS-IPs from human (H) and mouse (M) cardiac samples. Additional α-dystrobrevin isoforms (arrow heads) are detected in human cardiac lysates and DYS-IP.

The stoichiometry of syntrophins is affected by alternative splicing of α-dystrobrevin [28]. We therefore tested for tissue-specific association of α-dystrobrevin splice variants (α1-, α2-, and α3-) with cardiac and skeletal muscle dystrophin. Because of their high sequence identity, α-dystrobrevin splice variants could not be conclusively distinguished by LC-MS/MS. However, Western blot analysis with a pan α-dystrobrevin antibody allowed distinction of α-dystrobrevin variants based on molecular weight. Both α1- and α2-dystrobrevins were present in mouse cardiac and skeletal muscle DYS-IPs but they differed in their relative stoichiometry to dystrophin (Figure 4A). The stoichiometry of α2-dystrobrevin to dystrophin was similar between skeletal and cardiac muscle DYS-IPs (Figure 4A) in agreement with strong α2-dystrobrevin immunolabeling at the membrane of all cardiomyocytes (Figure 4B) and skeletal muscle fibers (Figure S2) where dystrophin is also expressed. By contrast, almost 5-fold less α1-dystrobrevin was associated with cardiac DYS-IPs compared to skeletal muscle (Figure 4A). Accordingly, α1-dystrobrevin was expressed at the sarcolemma of all myofibers in skeletal muscle (Figure S2) but showed only weak discontinuous membrane staining of a subset of cardiomyocytes (Figure 4B).

Western blot analysis also revealed that α3-dystrobrevin, while expressed in both mouse skeletal and cardiac muscle, strongly associates with dystrophin in the heart (Figure 4A). In skeletal muscle DYS-IPs, a faint but specific α3-dystrobrevin band could only be detected after long exposures suggesting that only a small pool of α3-dystrobrevin associates with full length skeletal muscle dystrophin. Interestingly, α3-dystrobrevin is the only splice variant that lacks the known dystrophin and syntrophin binding domains [29], [30] and associates with the DAPC via direct binding to the intracellular domain of β-sarcoglycan [31]. A prior study [9] and our own data (Figure 4C) indicate that β-sarcoglycan is preserved at the membrane of dystrophin-deficient cardiomyocytes. We therefore predicted that α3-dystrobrevin would be similarly unaffected in the *mdx* heart. Surprisingly, loss of dystrophin in *mdx* mice is accompanied by an almost complete loss of α3-dystrobrevin expression in the heart (Figure 4D). Among all three α-dystrobrevin splice variants, α3-dystrobrevin showed the most drastic decrease in expression in the *mdx* heart. These results confirm that α3-dystrobrevin is a member of the cardiac DAPC and further indicate that its expression in cardiac muscle is tightly dependent upon dystrophin, even in the presence of β-sarcoglycan at the membrane of *mdx* cardiomyocytes.

Finally, we confirmed by Western blot analysis that all three α-dystrobrevin isoforms interact with dystrophin in the human heart (Figure 4E). Interestingly, the antibody to α-dystrobrevin recognized additional bands in the human cardiac dystrophin IPs. Based on molecular weight, these additional bands likely correspond to the previously described human-specific α-dystrobrevin splice variants [32]. These results suggest that while the human cardiac DAPC is similar to the mouse, it is also more complex in its α-dystrobrevin composition.

### New Cardiac-specific Protein Associations of Dystrophin

**Figure 5:**
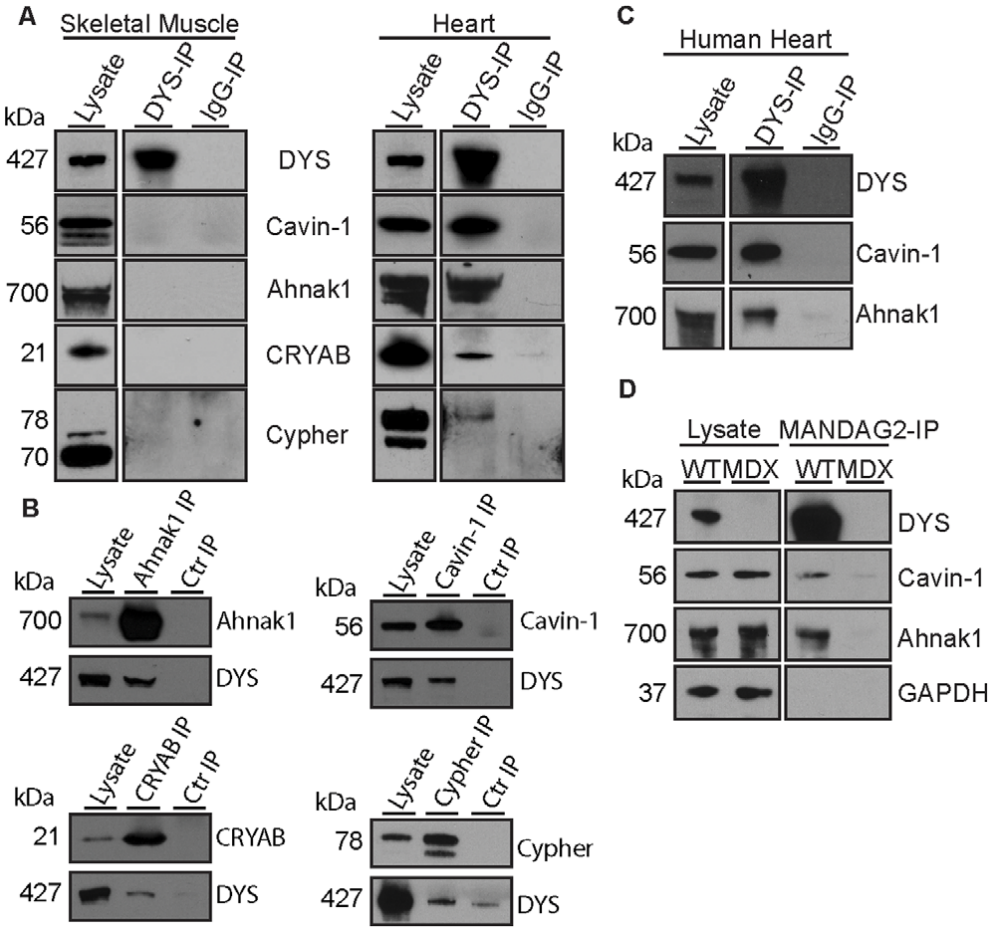
Novel cardiac-specific dystrophin-associated proteins. (**A**) Western blot analysis of Cavin-1, Ahnak1, CRYAB, and Cypher in mouse cardiac and skeletal muscle lysates, DYS-IPs and IgG-IPs. (**B**) Cavin-1 and Ahnak1 co-purify with dystrophin in human cardiac samples. (**C**) Western blot analysis of Cavin-1 and Ahnak1 in MANDAG2-IPs from wild type (WT) and *mdx* cardiac muscle. Expression levels of Cavin-1 and Ahnak1 are not decreased in *mdx* lysates compared to WT.

Our comparative LC-MS/MS analysis suggested novel cardiac-specific interactions of dystrophin with Cavin-1, Ahnak1, Cypher and CRYAB (Table 2) and these proteins were specifically and consistently detected by Western blot analysis in mouse cardiac but not skeletal muscle DYS-IPs (Figure 5A). Although these proteins are abundant in mouse muscle, they were not detected in IgG- or MDX-IPs by LC-MS/MS (Table 2) or Western blots (Figure 5A), with the exception of low amounts of CRYAB detected by Western blot after long exposure. Of note, only a fraction of the total pool of CRYAB and Cypher co-purified with dystrophin suggesting that these interactions are either weak or rare. The interactions of these cardiac proteins with dystrophin was further confirmed by reverse immunoprecipitation for each individual protein on cardiac mouse tissue (Figure 5B). No dystrophin was detected in the IgG matched controls except for trace amounts in the cypher immunoprecipitation.

Strong immunoreactive bands corresponding to Cavin-1 and Ahnak1 were consistently detected in cardiac DYS-IPs from the mouse (Figure 5A). We therefore asked whether these interactions were conserved in the human heart. Similar to the mouse, strong immunoreactive bands were detected in DYS-IPs from a human cardiac biopsy (Figure 5C). Densitometric analysis of the expression of Cavin-1 and Ahnak1 in *mdx* versus wild type mouse cardiac muscle did not reveal any significant change in overall expression. We therefore tested whether dystrophin was required for their association with the cardiac DAPC. We immunoprecipitated the DAPC from wild type and *mdx* mouse heart using the MANDAG2 antibody to β-dystroglycan, that is preserved at the membrane of *mdx* cardiomyocytes. Although MANDAG2 recognizes a region of β-dystroglycan involved in binding to dystrophin, it co-purified large amounts of dystrophin as well as Ahnak1 and Cavin-1 in wild type heart (Figure 5D). By contrast, Ahnak1 and Cavin-1 were absent from MANDAG2-IPs from *mdx* mouse heart. Therefore, Ahnak1 and Cavin-1 are part of the cardiac DAPC and this association is affected in the dystrophin-deficient heart.

## Discussion

The study of the interactome of dystrophin has been hampered by the large size of dystrophin and by the complexity of its direct and indirect interactions with extracellular, transmembrane, and intracellular proteins. Yet the identification of dystrophin-associated proteins is fundamental to our knowledge of the functions of dystrophin in healthy striated muscles and, by correlate, to our understanding of the molecular events that trigger cardiac and skeletal muscle disease in dystrophinopathies.

This study describes a rapid and simple protocol that allows for the high confidence and robust identification of proteins that interact either directly or indirectly with dystrophin within muscle tissues. Compared to previous studies [10], [33], this approach offers the advantage of specifically purifying dystrophin-containing complexes with the MANDYS1 antibody rather than using lectins that bind to multiple glycosylated proteins. We are also directly interrogating the entire protein eluate with minimal losses in sensitivity associated with gel separation of proteins. This resulted in impressive protein coverage, high confidence scores and reliable detection of all core DAPC members. This high level of sensitivity likely played a key role in our ability to identify with high confidence Cypher, Ahnak1, Cavin-1, and CRYAB as new cardiac-specific dystrophin-associated proteins. Indeed, our Western blot results indicate that these proteins are not abundant in dystrophin immunoprecipitations, possibly explaining why they have not been previously detected. Finally, we downsized the amount of starting material required to 50 mg of tissue. The ability to study the interactome of dystrophin from small amounts of tissue is not of trivial importance. It enables the study of DAPC composition by proteomics in the mouse, an animal model for many human muscle disorders [34], [35]. In combination with the spectral count analysis described here, this opens the door to future comparative studies in mouse models of muscular dystrophies aimed at understanding how disease-causing mutations may affect the composition of the DAPC. In addition, 50 mg is within the size range of a human tissue biopsy, suggesting that our protocol may be adapted in the future to directly study the DAPC in human muscles. Analysis of human cardiac samples may be particularly informative since our results with α-dystrobrevin suggest a higher complexity of the human cardiac DAPC compared to the mouse.

The successful comparison of affinity purified dystrophin protein complexes between cardiac and skeletal muscles required a tissue-specific background strategy and was further made possible by a multi-factor quantitative bio-informatics approach applied to spectral counts. From a biological standpoint, our approach provided a global yet detailed view of the DAPC in cardiac and skeletal muscle that clearly revealed differences specifically affecting the syntrophin-dystrobrevin sub-complex and its interaction with nNOS. The finding that nNOS is not part of the cardiac DAPC suggests that dystrophin is not involved in nNOS membrane localization in cardiomyocytes as it is in skeletal muscle fibers [26], [36]. This agrees with the reported interaction of nNOS with the ryanodine receptor in the heart and its spatial confinement to the sarcoplasmic reticulum [24] where dystrophin is absent. A link between cardiac dystrophin and nNOS has been postulated based on decreased nNOS activity in *mdx* hearts [37], [38] and on improved cardiac histopathology upon nNOS over-expression [39]. Our data favors a model where perturbations in cardiac nNOS activity are secondary to the loss of dystrophin, while in skeletal muscle deregulated nNOS function is a direct consequence of lack of dystrophin at the myofiber membrane [40], [41].

Our findings also raise the question of the identity of the proteins that interact with the cardiac syntrophin/dystrobrevin complex and the nature of the intracellular functions they may mediate. Given a general paucity of information on binding partners for syntrophins and dystrobrevins, we cannot at this time address the functional significance of the observed preferential inclusion of β2-syntrophin and α3-dystrobrevin in the cardiac DAPC. Interestingly, mice genetically engineered to lack α1-, α2- and α3-dystrobrevins show cardiomyocyte degeneration, inflammation and fibrosis indicating that α-dystrobrevins are important for cardiac integrity [42]. In addition, *in* *vitro* studies have indicated that all three syntrophins can interact with and may regulate the activity of the cardiac sodium channel Nav1.5 which is disrupted in the dystrophin-deficient heart [43].

Finally, our findings open the door to studies into the functional significance of the novel cardiac-specific association of dystrophin with Cavin-1, Ahnak1, Cypher and CRYAB. These novel associations suggest a direct link between dystrophin and two major cardiac systems that are disrupted in the dystrophin-deficient heart: the regulation of ion channels that initiate and pace contraction, and the sarcomeric apparatus that ultimately mediates cardiac contraction. Indeed, the majority of dystrophinopathy patients have arrhythmias, long QT syndrome and contractile dysfunction [7], and therefore overlap in phenotype with patients with mutations in cypher, CRYAB, Ahnak1, or Cavin-1 [17], [21], [22], [23], [44]. Therefore, our proteomics findings suggest a novel molecular link between these different cardiac diseases that warrants further investigation.

## Methods

### Human Ethics and Cardiac Biopsies

Human cardiac biopsy material was obtained with informed written consent from guardians on behalf of the minor participants involved in the study under our approved IRB protocol (IRB07-00225). Surgeries were performed at Nationwide Children’s Hospital. Discarded ventricular tissue from corrective cardiac surgery in infants (1 to 3 months old) diagnosed with Tetralogy of Fallot was used. Tetralogy of Fallot is a congenital heart defect that does not involve mutations in DAPC members and leads to an outgrowth of histologically normal ventricular tissue. Biopsies from two unrelated patients were analyzed in this study. For each patient, 100 mg of biopsy tissue were processed for one MANDYS1 IP and one Control IP.

### Animals

C57BL/6J, dystrophin-deficient *mdx^5cv^* (referred to as *mdx*), nNOS knock-out (KN1) [45] and dystrobrevin knock-out [42] mice between 12 and 20 weeks of age were used. Mice were provided with full access to food and water. Animal procedures were approved by the Institutional Animal Care and Use Committees at Nationwide Children’s Hospital or the University of Washington.

### Antibodies

Anti-dystrophin (MANDYS1), isotype-matched control (MW8), and anti-β-dystroglycan (MANDAG2) antibodies were produced in-house from hybridoma cell lines (DSHB; University of Iowa) and concentrated using the Amicon ultra-filtration cell (Millipore). Antibodies to DAPC members are: isoform specific anti- α1-, β1- or β2-syntrophin, and anti- α1- or α2-dystrobrevin antibodies [27], [30], [46]; pan anti-syntrophin (ab11425, Abcam); Manex1011B to dystrophin and MANDAG2 to β-dystroglycan (DSHB); clone IIH6C4 to α-dystroglycan (Upstate); anti-nNOS (#610308) and anti-α-dystrobrevin (#610766, BD Bioscience); anti-β-sarcoglycan (clone 5B1, Leica Microsystems).

### Immunoprecipitations

For mice, one quadriceps muscle and left and right cardiac ventricles were dissected per mouse, weighed and 100 mg of tissue were homogenized for protein extraction. This amount of tissue provided enough material for one experimental IP and one control IP from the same protein homogenate. Quadriceps muscles were chosen for analysis because of their mixed fiber type composition. For human patients, cardiac biopsies of ventricular tissue were obtained from 2 patients and 100 mg of each were homogenized. Tissues from different mice or patients were not pooled. Tissues were homogenized 1∶10 w/v in ice cold Buffer A (1% digitonin, 0.05% NP-40, NaCl 150 mM, Tris 50 mM, pH 7.4) with Complete Protease Inhibitors and PhosSTOP (Roche Diagnostics) using a polytron homogenizer (Power Gen 700, Fisher Scientific). Proteins were extracted on ice for 1 hr, centrifuged at 80,000×g for 30 min and supernatant was pre-cleared with protein G agarose beads (Invitrogen). Protein concentration was determined with the Dc Protein Assay (Bio-Rad). MANDYS1, MANDAG2, or MW8 control antibodies were incubated with Dynal protein G magnetic beads (Invitrogen; 1.2 µg antibody per 1 µl beads) in 100 mM sodium phosphate (pH 5.0) overnight at 4°C. Antibodies were cross-linked to the beads by incubation in 0.2 M Triethanolamine containing 20 mM dimethyl pimelimidate for 30 min at 20°C. Antibody conjugated beads were incubated with 2–5 mg protein at 4°C for 3 hr, washed in ice cold Buffer A without digitonin, and proteins were eluted in Laemmli Reducing Sample Buffer for Western blot analysis or in 2% SDS, 100 mM DTT for LC-MS/MS analysis.

For reverse immunoprecipitation, samples were prepared as above. Antibodies used were anti-Cavin-1 (ab48824, Abcam), anti-Ahnak1 (EM-09, Cedarlane), anti-CRYAB (ab13496, Abcam), and anti-Cypher (ab40840, Abcam). The controls for Ahnak and CRYAB immunoprecipitations used equal amounts of species matched MW8 antibody as previously mentioned. For Cavin-1 and Cypher immunoprecipitations, species matched, non-specific rabbit (#305-005-003, Jackson ImmunoResearch) and goat (#111-005-003, Jackson ImmunoResearch) antibodies were used, respectively.

### LC-MS/MS

Proteomic analysis was performed on 2 MDX-IPs (2 biological replicates), and 3 DYS-IPs and their corresponding 3 IgG-IPs (3 biological replicates) from quadriceps and cardiac muscle samples. Eluted proteins were chloroform/methanol precipitated, resuspended in 5X Invitrosol protein solubilizer (Invitrogen), diluted with 25 mM ammonium bicarbonate to a final volume of 1X Invitrisol. The proteins were then reduced with 10 µL DTT (5 mg/mL solution in 100 mM ammonium bicarbonate) and carbamidomethylated with 10 µL Iodoacetimide solution (15 mg/mL in 100 mM ammonium bicarbonate). Trypsin (in 50 mM ammonium bicarbonate) was added to the protein solution with an enzyme to substrate ratio of 1∶25 (w/w). Samples were incubated for 2 hr at 37°C before quenching by acidification. Capillary-liquid chromatography-nanospray tandem mass spectrometry was performed on a Thermo Finnigan LTQ orbitrap mass spectrometer (Thermo Fisher Scientific, San Jose CA) equipped with a microspray source (Michrom Bioresources Inc, Auburn, CA) operated in positive ion mode. Samples were loaded onto a precolumn Cartridge (Dionex, Sunnyvale, CA) and desalted with 50 mM acetic acid for 10 minutes, then separated on the capillary column (0.2×150 mm Magic C18AQ 3 µ 200A, Michrom Bioresources Inc, Auburn, CA) using an UltiMate™ 3000 HPLC system from LC-Packings A Dionex Co (Sunnyvale, CA). Mobile phases A and B were 0.1% formic acid in water and 0.1% formic acid in acetonitrile, respectively. Flow rate was 2 µl/min. Mobile phase B was increased from 2% to 50% in 250 min, then from 50% to 90% in 5 min, then kept at 90% for another 5 min The column was equilibrated at 2% of mobile phase B (or 98% A) for 30 min before the next sample injection. MS/MS data was acquired with a spray voltage of 2 KV and a capillary temperature of 175°C. The scan sequence of the mass spectrometer was based on the TopTen™ method. The full scan mass resolving power was set at 30,000 to achieve high mass accuracy MS determination. The CID fragmentation energy was set to 35%. Dynamic exclusion is enabled with a repeat count of 3 within 30 s, a mass list size limit of 200, exclusion duration of 350 s and a low mass width of 0.50 and high mass width of 1.50 Da.

### Peptide Sequence Analysis

The RAW data files collected on the mass spectrometer were converted to mzXML and MGF files by use of MassMatrix data conversion tools (version 1.3, http://www.massmatrix.net/download). Isotope distributions for the precursor ions of the MS/MS spectra were deconvoluted to obtain the charge states and monoisotopic *m/z* values of the precursor ions during the data conversion. Resulting.mgf files were searched using Mascot Daemon (version 2.3.2, Matrix Science, Boston, MA) against UniprotKBSwiss mouse database (version072711, 55,744 protein sequences). Trypsin was used as the enzyme and three missed cleavages were permitted. Considered variable modifications were oxidation (Met) and carbamidomethylation (Cys). The mass accuracy of the precursor ions was set to 10 ppm and the fragment mass tolerance to 0.5 Da. Accidental picking of one 13 C peak was included into the search. The significance identity threshold was set at p<0.05 for valid protein identification. False discovery rates (FDR) for peptide matches were estimated using the target-decoy search strategy [47], [48]. All reported results are for peptides with less than 5% FDR except for one (heart control 2, which was 8.1%). Proteins with a Mascot mowse score of 25 or higher containing a minimum of one unique peptide with a *-b* or *-y* ion sequence tag of five residues or better were accepted. However, identifications of proteins with one unique peptide were considered to be true positives only if: 1) the precursor ion has correct charge status and the mass accuracy is <10 ppm. 2) *b* and *-y* ion sequential tag of five or more residues were present following manual validation of MS/MS spectra.

### Label-Free Quantitation

Label free quantitation was performed using the spectral count approach [49], [50] in which the relative protein quantitation is measured by comparing the number of MS/MS spectra identified from the same protein in each of the multiple LC/MSMS datasets. To evaluate statistically significant differences in protein abundances, the MS/MS data for each treatment and its technical replicates were combined using an in-house developed application into parsimonious protein lists. Differential expression of protein between the IgG-IP and DYS-IP were determined by analysis of the spectral count data using the edgeR bioconductor package [15]. Peptide spectral counts were modeled as an overdispersed Poisson/negative binomial distribution in which an empirical Bayes procedure was used to moderate overdispersion across each protein. An exact text for overdispersed data was then used to assess difference in protein abundance [51].

### Immunolabeling

Heart and quadriceps were flash frozen in isopentane cooled in liquid nitrogen and 4–8 µm serial sections were cut. Cardiac ventricular myocytes were isolated from 12–16 week old mice [52] and fixed with 0.5% paraformaldehyde in PBS. Immunofluorescence labeling of tissue sections and cardiomyocytes was performed as described [53]. Images were captured using an Olympus BX61 microscope or a Leica TCS-NT confocal microscope.

### Immunoblots

Proteins separated on 4–12% gradient SDS-PAGE gels (Invitrogen) were transferred to nitrocellulose membranes (Whatman), blocked with 5% skim milk in 0.1% Tween 20/Tris-buffered saline and incubated with primary antibodies. Membranes were incubated with appropriate horseradish peroxidase-conjugated secondary antibodies (Jackson ImmunoResearch) and enhanced chemiluminescence reagents (Pierce). Signal was detected on X-Ray film (RPI).

### Densitometric Analysis

Protein band intensities from multiple non-saturated film exposures were quantified using ImageJ (NIH). Values in the linear range of pixel intensities were selected for quantifications. Samples to be compared (cardiac versus skeletal muscle, wild type versus dystrophin-deficient) were run side by side on the same gel. For immunoprecipitations, the membrane was cut at the 200 kDa marker to simultaneously probe for dystrophin (top portion) and syntrophins or dystrobrevins (bottom portion). Signal intensities were normalized to the dystrophin signal. For total protein lysates, membranes were probed for the protein (s) of interest then stripped and re-probed for GAPDH. Band intensities were normalized to the GAPDH signal. Blots from 3 biological replicates were analyzed by densitometry for each protein.

## Supporting Information

Figure S1 **Immunoprecipitation strategy and background reduction.** **(A)** Effect of antibody cross-linking to beads on the contamination of immunoprecipitation samples by immunoglobulins. Western blot analysis of indicated DAPC members and immunoglobulins present in dystrophin (DYS) immunoprecipitations using MANDYS1-conjugated beads that were pre-treated (+) or not (−) with a cross-linking agent. Antibody bands (IgG) obscure syntrophin (Syn) and β-dystroglycan (β-DG) detection in the absence of cross-linker. **(B)** Antibody cross-linking eliminates contamination of immunoprecipitated proteins by immunoglobulins. No large IgG bands are seen at 50 kDa and 25 kDa after Deep Purple total protein dye staining of SDS-Page gel. IP: proteins eluted following skeletal muscle dystrophin immunoprecipitation. L: Molecular weight ladder. **(C)** Experimental design. Proteins were extracted from quadriceps muscle from wild type (WT) and dystrophin-deficient mdx mice. The MANDYS1 antibody to dystrophin and an isotype matched control antibody (IgG Control) were cross-linked to magnetic G-protein beads for immunoprecipitations. As an additional control for non-specific protein binding, MANDYS1 immunoprecipitations were also carried out on muscle tissue extracts from mdx mice. Bound proteins were eluted and processed for LC-MS/MS or immunoblots.
(TIF)

Figure S2 **Immunolabeling for dystrophin, nNOS, syntrophins and dystrobrevins in wild type skeletal muscle.** Immunolabeling of quadriceps muscle sections for dystrophin (DYS), α1-syntrophin (α1-Syn), β1-syntrophin (β1-Syn), nNOS, α1 and α2-dystrobrevin (DTNA1, DTNA2) (red) and DAPI (blue). Scale bar = 50 mm.
(TIF)

Figure S3 **Mass spectra for DAPC members identified by a single peptide in cardiac DYS-IPs.** Peaks matching the theoretical fragments ions are labeled as y ions (green), b ions (red) and b* ions (blue). As shown in the spectra, precursor ions have correct charge status and the mass accuracy is <2.5 ppm; the presence of b and −y ion sequential tag of five or more residues were also observed in the MS/MS spectra of these unique peptides. b* ions = nb-H20.
(TIF)

Figure S4 **Mass spectra for DAPC members identified by a single peptide in skeletal muscle DYS-IPs.** Peaks matching the theoretical fragments ions are labeled as y ions (green), b ions (red). As shown in the spectra, precursor ions have correct charge status and the mass accuracy is <4.5 ppm; the presence of b and −y ion sequential tag of five or more residues were also observed in the MS/MS spectra of these unique peptides.
(TIF)
