# Supplementary figures and images for: Proteomic Analysis Reveals New Cardiac-Specific Dystrophin-Associated Proteins
Source: PLoS One. 2012 Aug 24;7(8):e43515. doi: 10.1371/journal.pone.0043515 (PMC3427372; doi:10.1371/journal.pone.0043515)

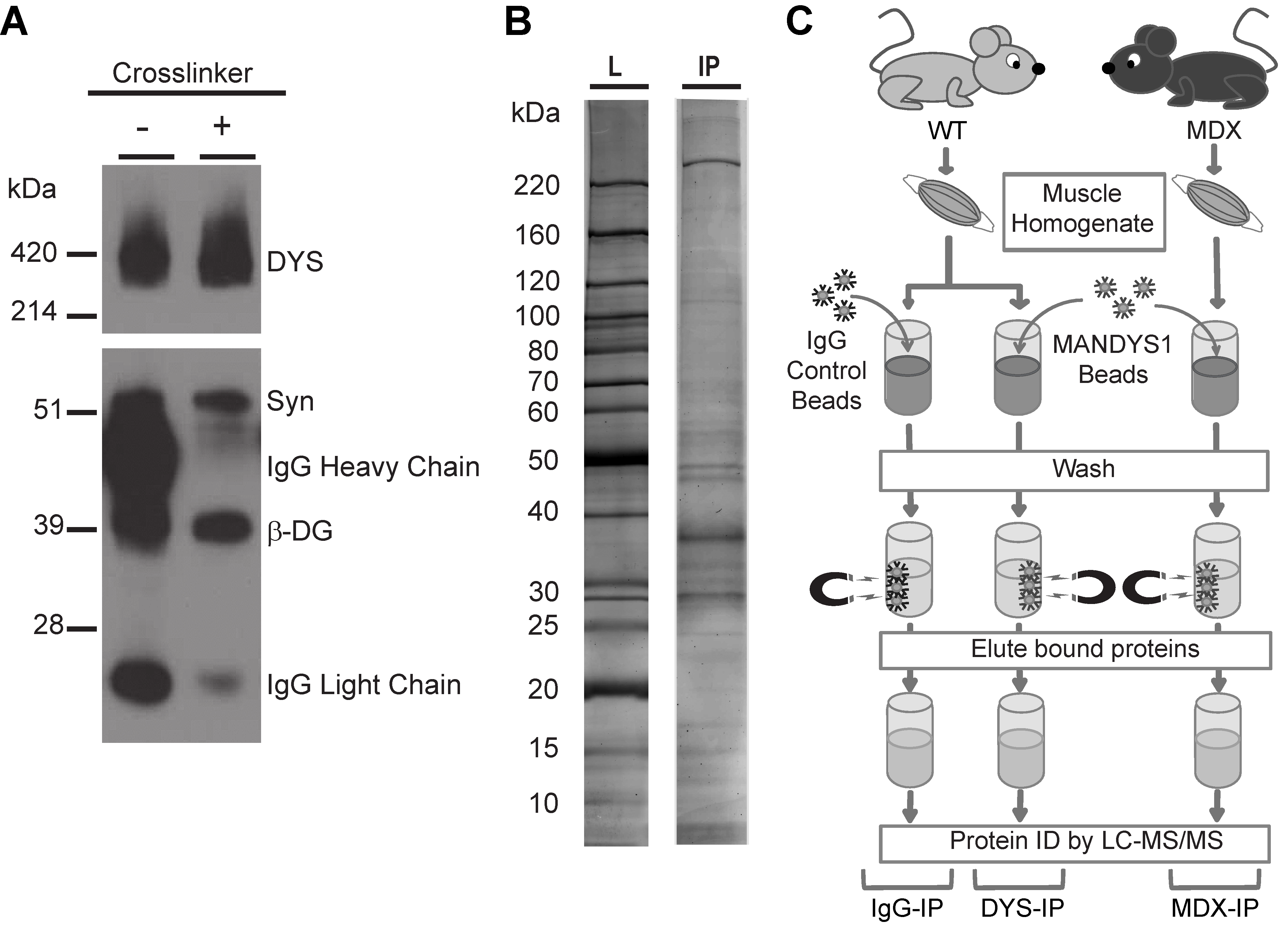

Supplement: Figure S1 — Immunoprecipitation strategy and background reduction. (A) Effect of antibody cross-linking to beads on the contamination of immunoprecipitation samples by immunoglobulins. Western blot analysis of indicated DAPC members and immunoglobulins present in dystrophin (DYS) immunoprecipitations using MANDYS1-conjugated beads that were pre-treated (+) or not (−) with a cross-linking agent. Antibody bands (IgG) obscure syntrophin (Syn) and β-dystroglycan (β-DG) detection in the absence of cross-linker. (B) Antibody cross-linking eliminates contamination of immunoprecipitated proteins by immunoglobulins. No large IgG bands are seen at 50 kDa and 25 kDa after Deep Purple total protein dye staining of SDS-Page gel. IP: proteins eluted following skeletal muscle dystrophin immunoprecipitation. L: Molecular weight ladder. (C) Experimental design. Proteins were extracted from quadriceps muscle from wild type (WT) and dystrophin-deficient mdx mice. The MANDYS1 antibody to dystrophin and an isotype matched control antibody (IgG Control) were cross-linked to magnetic G-protein beads for immunoprecipitations. As an additional control for non-specific protein binding, MANDYS1 immunoprecipitations were also carried out on muscle tissue extracts from mdx mice. Bound proteins were eluted and processed for LC-MS/MS or immunoblots. (TIF) [file pone.0043515.s001.tif]

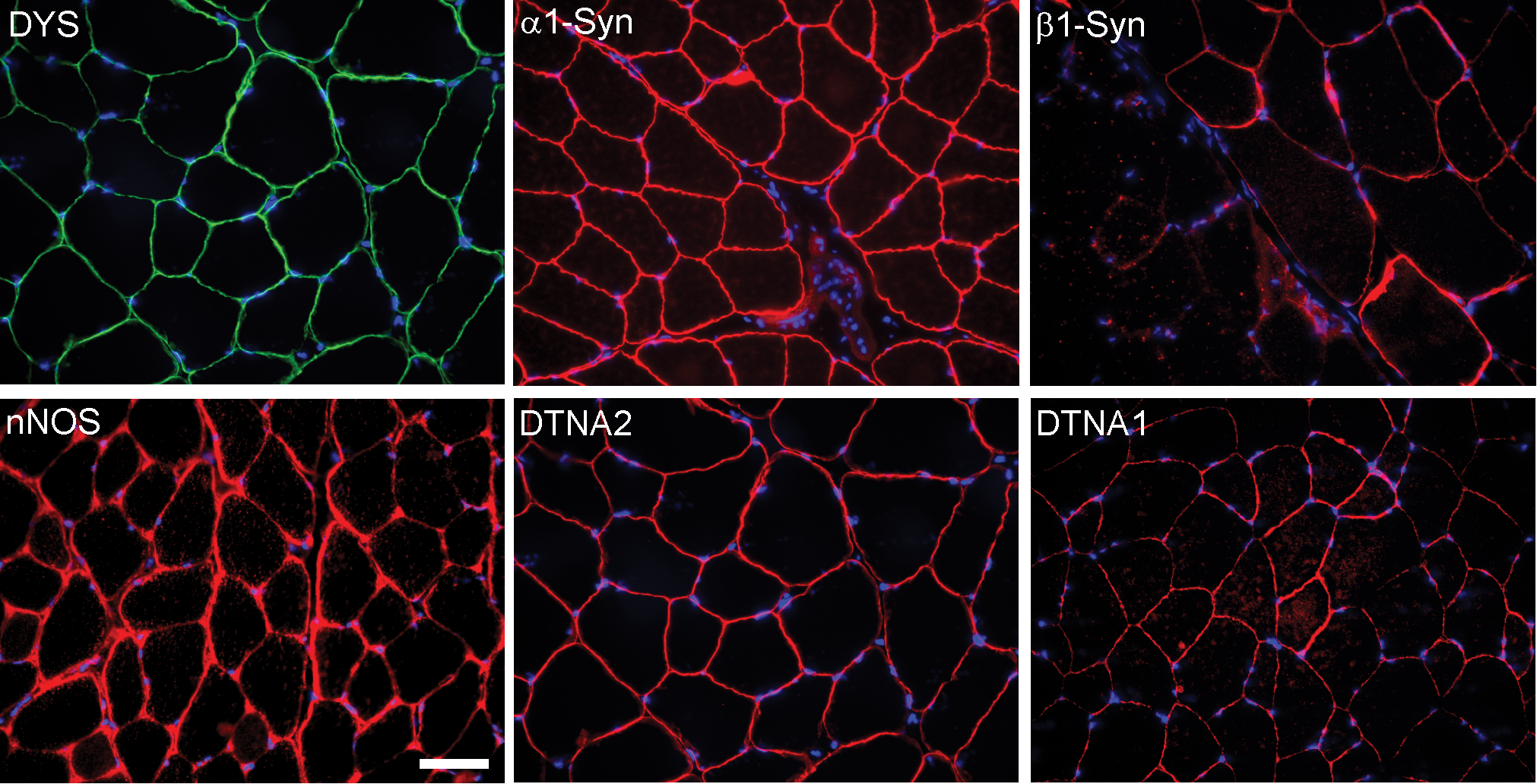

Supplement: Figure S2 — Immunolabeling for dystrophin, nNOS, syntrophins and dystrobrevins in wild type skeletal muscle. Immunolabeling of quadriceps muscle sections for dystrophin (DYS), α1-syntrophin (α1-Syn), β1-syntrophin (β1-Syn), nNOS, α1 and α2-dystrobrevin (DTNA1, DTNA2) (red) and DAPI (blue). Scale bar = 50 mm. (TIF) [file pone.0043515.s002.tif]

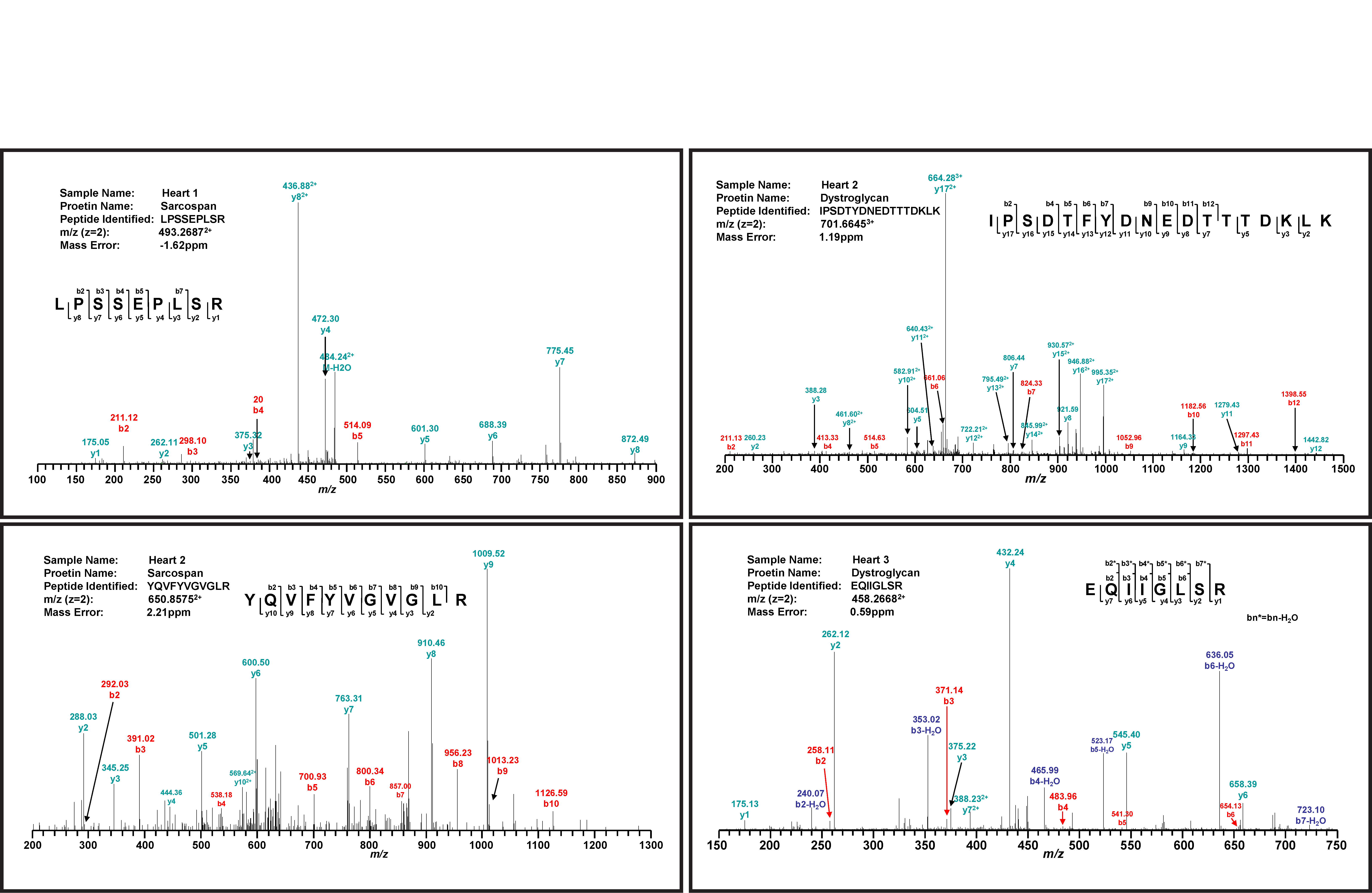

Supplement: Figure S3 — Mass spectra for DAPC members identified by a single peptide in cardiac DYS-IPs. Peaks matching the theoretical fragments ions are labeled as y ions (green), b ions (red) and b* ions (blue). As shown in the spectra, precursor ions have correct charge status and the mass accuracy is <2.5 ppm; the presence of b and −y ion sequential tag of five or more residues were also observed in the MS/MS spectra of these unique peptides. b* ions = nb-H20. (TIF) [file pone.0043515.s003.tif]

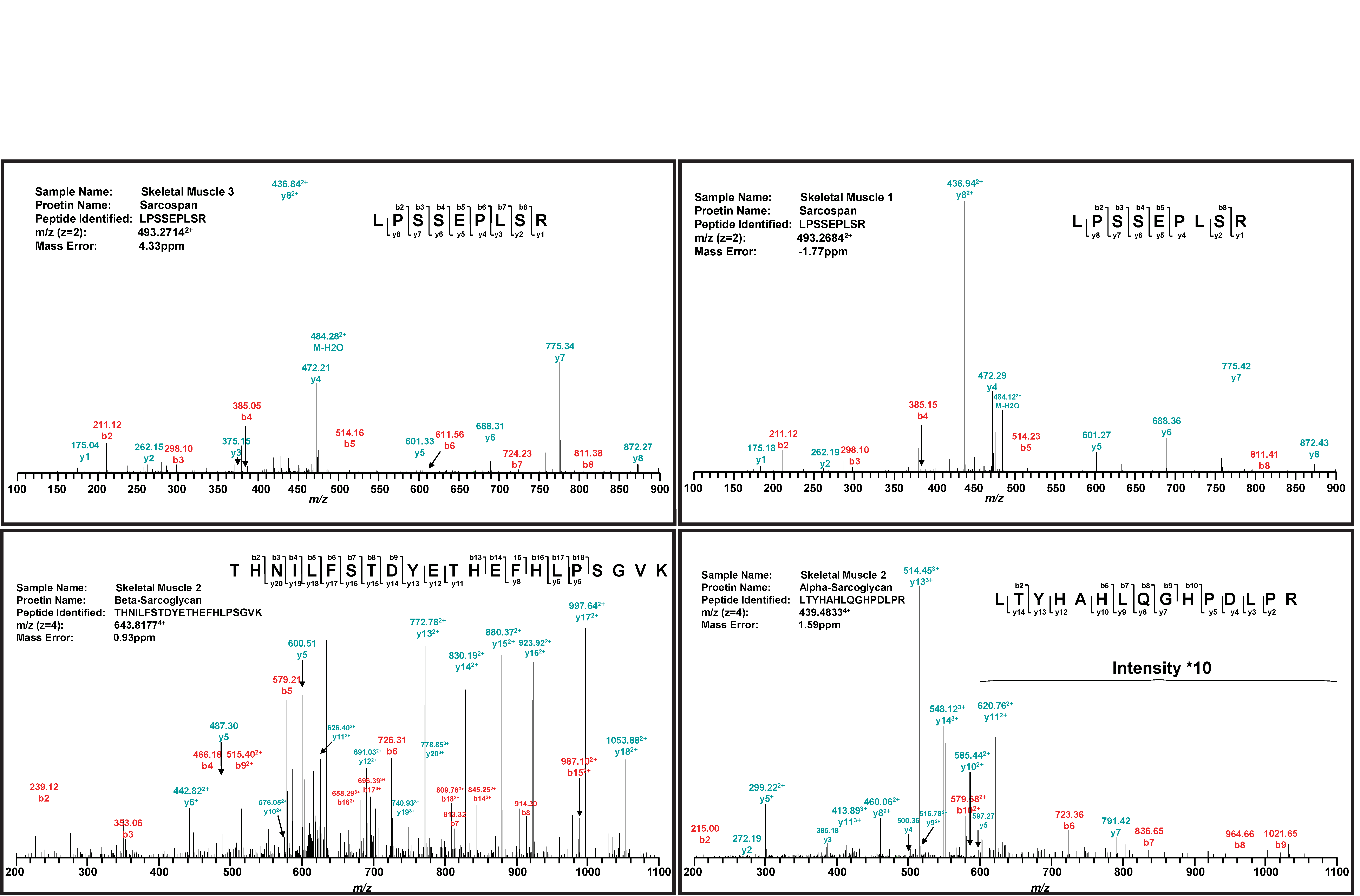

Supplement: Figure S4 — Mass spectra for DAPC members identified by a single peptide in skeletal muscle DYS-IPs. Peaks matching the theoretical fragments ions are labeled as y ions (green), b ions (red). As shown in the spectra, precursor ions have correct charge status and the mass accuracy is <4.5 ppm; the presence of b and −y ion sequential tag of five or more residues were also observed in the MS/MS spectra of these unique peptides. (TIF) [file pone.0043515.s004.tif]
